# Feeling stressed and depressed? A three-wave follow-up study of the beneficial effects of voluntary work

**DOI:** 10.1016/j.ijchp.2022.100363

**Published:** 2022-12-21

**Authors:** Yannick Griep, Linda Magnusson Hanson, Constanze Leineweber, Sabine A.E. Geurts

**Affiliations:** a Behavioural Science Institute, Radboud University, Nijmegen, the Netherlands; b Stress Research Institute, Stockholm University, Stockholm, Sweden

**Keywords:** Stress, Depression, Anti-depression treatment, Volunteering, Follow-up study

## Abstract

While symptoms of stress are a major risk factor in the onset of depressive symptoms and major depression, a better understanding of intervening mechanisms in breaking down this positive association is urgently required. It is within this literature that we investigate (1) how symptoms of stress are associated with depressive symptoms and the onset of major depression, and (2) the buffering effect of hours spent on voluntary work on the stress-depression relationship. Using 3-wave longitudinal data, we estimated a direct and reverse auto-regressive path model. We found both cross-sectional and longitudinal support for the positive association between symptoms of stress and depressive symptoms. Next, we found that individuals who experienced more symptoms of stress at T1, T2, and T3 were 1.64 (95%CI [1.46;1.91]), 1.49 (95%CI [1.24;1.74]), and 1.40 (95%CI [1.21;1.60]) times more likely to be prescribed an anti-depression treatment at T3, respectively. Moreover, we found that the number of hours spent volunteering mitigated the (1) longitudinal—but not cross-sectional—stress-depression relationship, and (2) cross-sectional—but not the longitudinal—association between symptoms of stress at T3 and the likelihood of being prescribed an anti-depression treatment. These results point toward the pivotal role of voluntary work in reducing the development of depressive symptoms and major depression in relation to the experience of symptoms of stress.

## Introduction

Major depression is considered the second most burdensome diseases in developed countries with considerable psychological suffering, morbidity, and mortality (López et al., 2006; Menken et al., 2000). Estimates suggest that being diagnosed with depression—a diagnosis based on the presentation of depressive symptoms such as feeling blue, lethargy, and excessive worrying—has a lifetime prevalence of 17% (Kessler et al., 2008). A plethora of work has attempted to better understand the role of symptoms of stress as a risk factor in the onset of depressive symptoms and the ultimate development of major depression (for a meta-analysis see Hosang et al. 2014). Traditionally this relationship is being understood with reference to the accumulation of symptoms of stress over time, until it spills over into the development of depressive symptoms and major depression. Whereas these meta-analytical studies are extremely valuable in our understanding of the aetiology of major depression, a better understanding of intervening mechanisms in breaking down this positive association between symptoms of stress and depression is urgently required. It is within this desire to push the field forward that we introduce voluntary work—that is (1) activities performed out of free will, (2) without receiving remuneration, (3) in a formal organization (i.e., society, relief organization, religious organization, political party or non-profit organization), and (4) benefiting others (Snyder & Omoto, 2008)—as a potential mitigating factor of the positive stress-depression relationship. We argue that the enactment of voluntary work—through its ability to provide (1) greater social interaction and social support, (2) greater autonomy, role accumulation, and identity, and (3) greater reinforcement and usage of one's skills and a sense of purpose—holds the potential to moderates the positive relationship of symptoms of stress with depressive symptoms and major depression.

A very small body of extant literature has focused on the potential stress-buffering effects of voluntary work (for the limited number of studies see Carr et al. 2018; Greenfield & Marks 2004; Han et al. 2018; Li 2007). These studies largely found support for the assumption that voluntary work weakens the link between stressful life experiences and feelings of loneliness (i.e., a single indicator of depressive symptoms; Carr et al., 2018), feelings of purpose of life (i.e., a single indicator of depressive symptoms; Greenfield & Marks, 2004), feelings of self-efficacy (i.e., a single indicator of depressive symptoms; Li, 2007), and general negative affect on days when one volunteered (i.e., an indicator of stress reactivity and not an indicator of depressive symptoms; Han et al., 2018). There are two major problems associated with these studies. First, these studies focus on self-reported single indicators of depressive symptoms, rather than on a validated set of depressive symptoms. In our study, we included both a validated multi-item scale to measure depressive symptoms and being prescribed an antidepressant following a medical diagnosis as a more objective indicator of major depression. Second, most of these studies were methodologically limited by the lengthy observational intervals (e.g., up to 5-year intervals between two subsequent waves of data collection), cross-sectional data (i.e., the direction of the effect cannot be investigated), and social selection bias. The current study overcomes these shortcomings by conducting a 3-wave, 2-year interval, study among a nationally representative longitudinal cohort of the Swedish population. In conducting this replication, while improving upon these methodological and conceptual issues, we are hopeful that the insights obtained from this study support a win-win-win proposition for the individual, the organizations in which these individuals complete their voluntary activities, and the society as a whole. Specifically, we hope to demonstrate the role of voluntary work in relation to breaking down the positive relationship between symptoms of stress and both the short- and long-term development of depressive symptoms and major depression following medical diagnosis.

### The stress-depression relationship - symptoms and diagnosis

There is a wealth of evidence supporting the positive association between symptoms of stress and the experience of depressive symptoms and the development of major depression (for meta-analyses see Burk et al. 2005; Feiss et al. 2019; Hosang et al. 2014; LeMoult et al. 2020). In the current study we aim to replicate and extent these findings by focusing on the relationship between symptoms of stress and depressive symptoms and major depression. Symptoms of stress This association can be understood with reference to the Stress Reaction Model (cross-sectional relationships) and the Accumulation Model (longitudinal relationships) of stress (Ford et al., 2014; Pindek et al., 2019; Zapf et al., 1996).

According to the premise of the Stress Reaction Model (Ford et al., 2014; Pindek et al., 2019; Zapf et al., 1996), the result of exposure to symptoms of stress increases strain responses in the form of various psychological, physiological, and behavioral outcomes, including depressive symptoms and major depression (de Jonge & Kompier, 1997; Hammen, 2005). This forms the basis for a positive *cross-sectional relationship* between symptoms of stress and depressive symptoms. However, an assumption of this model is that once symptoms of stress are removed, there is an improvement in psychological functioning. *The longitudinal relationship*, on the other hand, can be understood with reference to the Accumulation Model. In line with this model, scholars (Ford et al., 2014; Pindek et al., 2019; Zapf et al., 1996) assume that depressive symptoms and major depression are the result of an accumulation or extended exposure to a stressor, even when the stressor has been removed or its effect is more distal. There is indeed empirical evidence suggesting that both the experience of symptoms of stress at a single point in time (evidence for the cross-sectional relationship; e.g., Cheung & Yip 2015; Debowska et al. 2020; Jones-Bitton et al. 2020; Sameer et al. 2020; Shen et al. 2014; Varma et al. 2021) and the accumulation of symptoms of stress over time (evidence for the longitudinal relationship; e.g., Gao et al. 2020; Hosang et al. 2010, 2012a; Huang et al. 2018; Paulson 2022; Planchuelo-Gómez et al. 2020; Probst et al. 2020) may trigger depressive symptoms and major depression. A further investigating of the association between symptoms of stress and depressive symptoms and major depression has demonstrated that symptoms of stress are associated to the onset, relapse, and exacerbation of depressive symptoms and major depression in a causal way (e.g., Farmer & McGuffin 2003; Hosang et al. 2012b; Huckins et al. 2020; Maciejewski et al. 2001; Ridley et al. 2020). In sum, this leads us to hypothesize that symptoms of stress are positively associated with (1) depressive symptoms at the same point in time and over time, and (2) major depression over time.

Hypothesis 1 *Symptoms of stress are positively associated with (1) the experience of depressive symptoms at the same point in time (cross-sectional; H1a), (2) the experience of depressive symptoms over time (longitudinal; H1b), and (3) the likelihood of being diagnosed with major depression at the end of the study, thus at T3 (longitudinal; H1c).*

### Breaking the stress-depression relationship: the mitigating role of voluntary work

Scholars have paid considerable attention to the robust well-being and health benefits associated with voluntary work (for a review see Anderson et al. 2014; see also Bowe et al. 2022; Burr et al. 2011; Griep et al. 2015, 2017; Kamerāde & Bennett 2018; Li & Ferraro 2005; Mak & Fancourt et al. 2022; O'Brien et al. 2010; Pillemer et al. 2010; Russell et al. 2022). Overall, these studies demonstrate that individuals who volunteer seem to be in good physical health, have a lower risk of being diagnosed with hypertension later in life, are less likely to smoke and consume alcohol, are more satisfied with their life and suffer less from mental health complaints, psychological distress, and depressive symptoms and major depression. The above-described positive well-being and health consequences of volunteering are commonly explained by the satisfaction of basic psychological needs offered by voluntary work which has been argued to be among the most important factors that influence the stress-buffering process (Anderson et al., 2014; Brown & Brown, 2017; Brown & Okun, 2014; Inagaki, 2018; Pilkington et al., 2012), akin to the protective benefits of generosity underlying voluntary work (e.g., Hansen et al. 2017; Koenig et al. 2014; Pearce et al. 2015; Rodebaugh et al. 2016).

It should hence come as no surprise that scholars have found *cross-sectional and longitudinal* support for the argument that volunteering seems to alleviate the experience of symptoms of stress and positively affect one's psychological health (Musick & Wilson, 2003), which may have downstream effects on psychological indicators such as reduced psychological distress (Weinstein et al., 2016), reduced depressive symptoms (Kranabetter & Niessen, 2019; Lu et al., 2011; Talley et al., 2010), reduced depressive mood (van Hooff et al., 2016), and reduced major depression (Auclair-Pilote et al., 2021; Wei et al., 2005). Hence, it could be argued that when one experiences symptoms of stress but engages in voluntary activities, the symptoms of stress are less likely to trigger depressive symptoms at the same point in time and over time,and the development of major depression over time.

Hypothesis 2 *Enactment of voluntary work mitigates the positive relationship between symptoms of stress and (1) depressive symptoms at the same point in time (cross-sectional; H2a), (2) depressive symptoms over time (longitudinal; H2b), and (3) the likelihood of being diagnosed with major depression at the end of the study (longitudinal; H2c) in such a way that the association of symptoms of stress with depressive symptoms and the likelihood of being diagnosed with major depression will be weaker when voluntary work is high.*

## Material and methods

### Procedure

We conducted a 3-wave, with 2-years intervals, follow-up study among Swedish individuals using survey data from the to us available 2012 (T1), 2014 (T2), and 2016 (T3) waves of the Swedish Longitudinal Occupational Survey of Health (SLOSH); a nationally representative longitudinal cohort survey of the Swedish population. The SLOSH project is carried out within the framework of Stockholm Stress Centre, a FAS centre of excellence (grant #2009-1758). SLOSH and register linkages are covered by existing approvals from the Regional Research Ethics Board in Stockholm (Dnr, 2006/158-31; 2008/240-32 extended 2008-07-01; Dnr, 2008/1808-32; Dnr, 2009/337-32; Dnr, 2009/493-31/3; and Dnr, 2010/0145-32) and earlier from the research ethics committee at Karolinska Institutet (1992-09-21, Dnr 92-198, extended 2000-11-15, same Dnr, plus 2003-03-10, Dnr 03-125). SLOSH is furthermore approved internally by Statistics Sweden (SCB # 24/9784/2001, # 115894/820137-8, and # 858758-6/198 633) and by the National Board of Health and Welfare (SoS). This specific study was approved by the ethics board (ERB-17-0547).

Below we explain the procedure of the SLOSH data collection in detail (see Magnusson Hanson et al. 2018 for the cohort profile of the SLOSH study). SLOSH was initiated in 2006 as a follow-up of the cross-sectional Swedish Work Environment Survey (SWES). The SLOSH cohort thus far comprises participants from the SWES 2003, 2005, 2007, 2009 and 2011 surveys. The SWES participants are in turn sampled from the Labour Force Survey (LFS), which is conducted biennially by Statistics Sweden (SCB). Usually more than 20000 individuals, stratified by county, sex, citizenship and inferred employment status, are randomly drawn from the entire Swedish population to represent a nationally representative survey of the Swedish working population. These people are then contacted by telephone, from among whom a random sub-sample of gainfully employed people, 16-64 years of age, are sent self-completion SWES questionnaires. The number of participants who participated in the SWES have varied over the years: 9214 in 2003, 9703 in 2005, 7729 in 2007, 6354 in 2009 and 7926 in 2011, representing about 50-64% of the individuals invited to LFS.

**Fig. 1:**
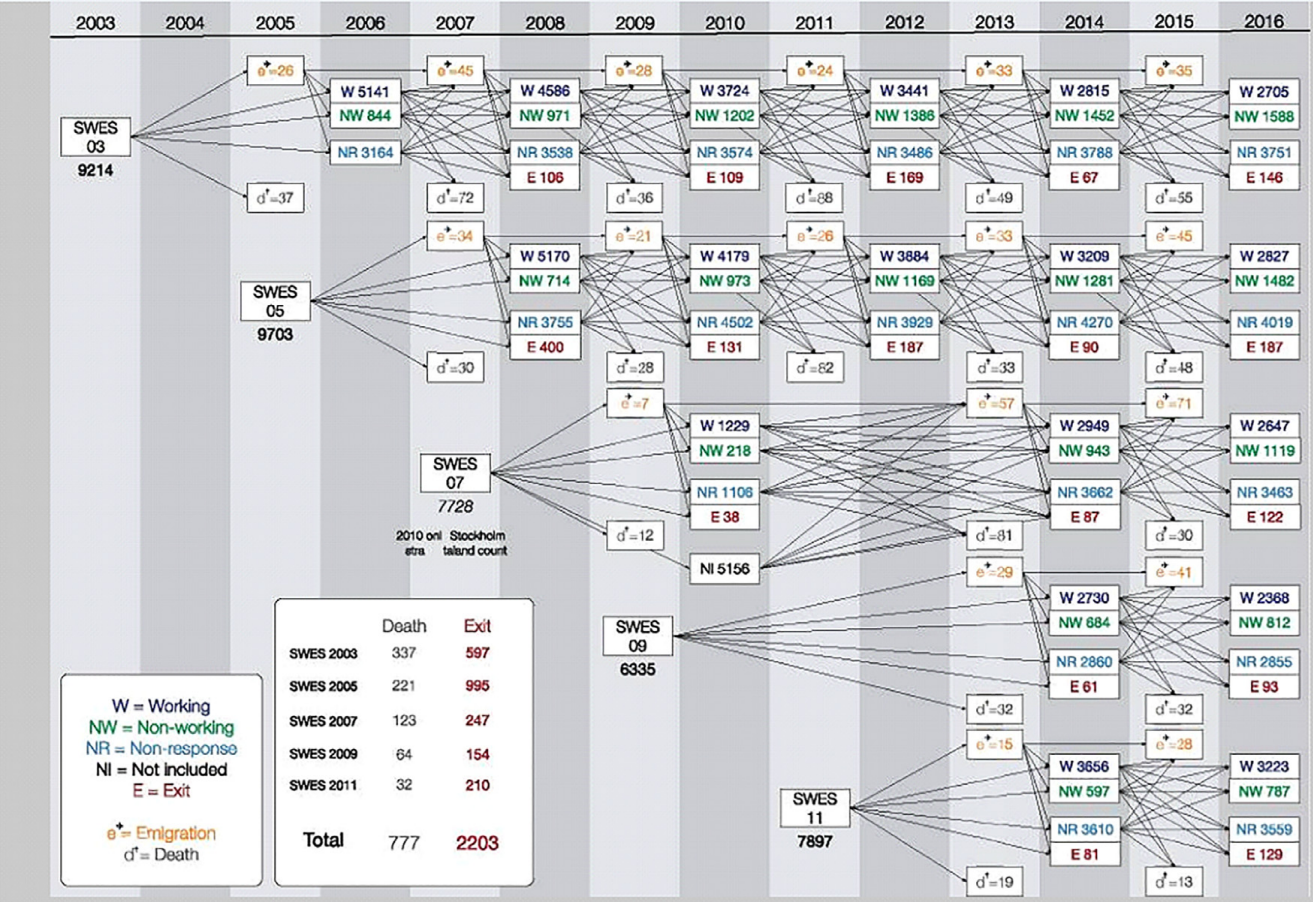
SLOSH and SWES study design and data collection for the period of 2003 to 2006 (SWES) and 2006 to 2016 (SLOSH). The figure also illustrates the number of respondents to the different versions of the questionnaire (W = “Working” and thus those individuals in paid work for 30% or more of their time during the past 3 months, NW = “Non-working” and thus those individuals in paid work < 30% of their time during the past 3 months) and the number of people exiting the study (NR = “non-response”; NI = “not included for various reasons such as not eligible for participation because of unknown address; E = “declined further participation”), emigrated (“e”) or had died (“d”) each wave (see also Magnusson Hanson et al. 2018).

As previously mentioned, the first SLOSH wave was collected in 2006 when the SWES respondents from 2003 were reapproached. In 2008, all eligible respondents to SWES 2003 and 2005 were contacted for a second time as part of the second SLOSH wave. In 2010, all eligible respondents from SWES 2003 and 2005 were contacted again for a third or second time, respectively, akin to SWES 2007 respondents (Stockholm and Västra Götaland regio) as part of the third SLOSH wave. In 2012, all eligible participants of SWES 2003 and 2005 were invited to participate for a fourth or third time, respectively as part of the fourth SLOSH wave. In 2014 and 2016, all participants in SWES 2003 and 2005, as well as 2007, 2009 and 2011, were invited as part of the fifth and sixth SLOSH wave. To further facilitate the interpretability of the recruitment and attrition across SLOSH waves, we included Fig. 1 which visually presents the data collection according to the baseline SWES cohort.

Those who were invited to take part in the study (see Fig. 1) were asked to return a questionnaire by means of a pre-stamped envelope. We provided written information about the purpose of the study, the discretionary nature of participation, the confidential treatment of the data, and the possibility to withdraw from the study. We requested respondents, who were willing to take part in the study, to return a questionnaire by means of a pre-stamped envelope. All data collection was carried out by Statistics Sweden (SCB). Each inhabitant in Sweden has a unique 10-digit personal identification number. This identification number is available to SCB and makes it possible to connect respondents’ answers from the SWES to registry data such as the Swedish National Prescribed Drug Register which contains data on dispensed out-patient prescriptions at all Swedish pharmacies following a medical diagnosis. When SWES respondents are asked to participate in SLOSH, the the Stress Research Institute prepares the questionnaires which are then send out by SCB. The questionnaires are sent back to SCB who scans the data and prepares the data set. Before providing the data to the authors, the personal identification number is replaced by a study specific identification number. SCB saves a key between the personal identification number and the questionnaire specific identification number to enable follow-up surveys. No incentives are used. To the best of our knowledge, the data collection of SCB is free of bias because SCB strives to get a representative sample of the Swedish working population.

### Participants

Of the 8484 individuals who could have completed the SLOSH waves, 6542 individuals (77.11% response rate) returned a questionnaire at T1, 6797 individuals (80.12% response rate) returned a questionnaire at T2, and 3987 individuals (46.99% response rate) returned a questionnaire at T3. Although we did not delete any respondents from the datafile prior to the below-described analytical approach, 2769 individuals completed at least two consecutive waves of data collection and were included in the analyses. Moreover, we conducted a logistic regression analysis to test whether participation at all times versus dropout (dropout = 1 versus non-dropout = 0) at any point in time could be predicted by our study variables and control variables. We found that dropout between different waves was lower among older respondents (OR = -.03, *p* < .001), and higher among male respondents (OR = .03, *p* < .001). However, none of the study variables predicted dropout.

At baseline (T1), our participants were on average 60.49 years old (*SD* = 11.42), 56.70% were female, 67.40% had a full-time contract, 36.20% had obtained a university degree, 41% had a higher educational degree, and 22.80% had a high school degree. Those who volunteered did this on average 5.97 h (*SD* = 7.27; range = 98 h) per week in 2012, 5.77 h (*SD* = 6.19; range = 69 h) per week in 2014, and 5.78 h (*SD* = 6.14; range = 80 h) per week in 2016.

### Measures

*Voluntary work* was measured with a single item used by previous studies in the field of (Occupational) Health Psychology and Clinical Psychology (Griep et al., 2015, 2017; Kim & Pai, 2010). The specific question is the following: “How many hours in an average week did you spend on voluntary (unpaid) work in, for example, a society, relief organization, religious organization, political party or non-profit organization?”. This single item measure is in line with well-established surveys (see Fisher et al. 2004) tapping into time spent volunteering such as the Time Use Surveys (TUS), Voluntary Work Surveys (VWS), World Values Surveys (WVS), and the American Time Use Survey (ATUS), akin to meta-analytical research into voluntary work (Niebuur et al., 2018). The types of voluntary activities are in line with the definition provided in the introduction and the TUS, VWS, WVS, and the ATUS list of categories of voluntary activities^1^. While zero represents no voluntary work, figures above zero (range of mean values = 5.77 – 5.97; range of SD values = 6.14 – 7.27) indicate the specific number of hours a respondent spent on voluntary work each week. We consequently used the number of hours spent volunteering (i.e., a measurement of exposure to volunteering ranging from zero—no exposure to voluntary work—to the maximum number of hours spent volunteering during a specific wave) as our moderator.

*Symptoms of stress* were measured with an 8-item stress scale (Magnusson Hanson et al., 2018), reflecting the core symptoms of long-term stress (e.g., difficulties relaxing, restless). All eight items make up a single stress factor (range of mean values = 1.75 – 1.77; range of SD values = .45 – .50). Respondents rated the extent to which they experienced these symptoms on a 4-point Likert scale ranging from ‘not at all’ (1) to ‘nearly all the time’ (4) (α_2012_=.90; α_2014_=.90; α_2016_=.89).

*Depressive symptoms* were measured with the 6-item symptom checklist for core depression (Magnusson Hanson et al., 2014), reflecting the core complaints and symptoms of depression (e.g., lethargy, feeling blue). All six items make up a single depressive symptoms factor (range of mean values = 1.69 – 1.76; range of SD values = .83 – 1.06). Respondents rated the extent to which they felt troubled by these symptoms on a 5-point Likert scale ranging from ‘not at all’ (1) to ‘very much’ (5) (α_2012_=.92; α_2014_=.91; α_2016_=.87).

*Antidepressant data* was retrieved from the Swedish National Prescribed Drug Register; a patient-based register that contains data on dispensed out-patient prescriptions at all Swedish pharmacies following a medical diagnosis. We extracted all redeemed prescriptions used in the *treatment of depression* (NO6A category) from 2010 until 2016 for all respondents. We focused on the likelihood of being prescribed an antidepressant. To capture this likelihood, we created a dummy variable with value “one” when respondents were prescribed an antidepressant (21.40%) and value “zero” when respondents were not prescribed an antidepressant (78.60%).

*Control variables* were included based on the proposed influence of age, gender, general health, and socio-economic status (as a combination of education and income as per the American Psychology Association) on depression. *Age* was measured in years. *Gender* was measured by asking respondents to indicate whether they were male (coded -1) or female (coded 1). *General self-rated health* was measured by asking respondents to rate their general state of health on a single item using a 5-point Likert scale ranging from ‘very poor’ (1) to ‘very good’ (5). *Education* was measured by asking respondents to indicate their highest obtained degree: university degree (coded as 1) versus all others (coded as -1), higher educational degree (coded as 1) versus all others (coded as -1), high school degree (coded as 1) versus all others (coded as -1). *Income* was measured as the mean combined family income in Swedish Krona.

### Analysis

We conducted an auto-regressive path analysis in Mplus version 8.3 (Muthén & Muthén, 2018) to test our hypotheses. Path analysis is an extension of conventional regression analysis because it allows for the simultaneous estimation of path coefficients from symptoms of stress, and its interaction with voluntary work, to depressive symptoms and being prescribed an antidepressant. (Lleras, 2005). To model change in the outcome, we included the outcome variable measured at an earlier point in time as a control variable. By doing so, changes in the outcome variables are not influenced by significant baseline differences or by previous measurements of the same variable at an earlier point in time. In this auto-regressive path model, we allowed for (1) all outcome variables to be correlated, (2) the control variables at T1 to be correlated with the outcome variables at T1, T2, and T3, and (3) all outcome variables at T2 and T3 to be correlated with themselves at T1 and T2, respectively. In the above-described analytical approach, we used Bayesian estimation methods. Although Bayesian analysis is relatively new in occupational health research, it proves to be especially suitable when dealing simultaneously with continuous (e.g., symptoms of stress) and binary (i.e., anti-depressant treatment) data, or when dealing with models that are otherwise hard to fit due to their complexity (for a detailed discussion of Bayesian analysis see Lewis et al. 2021).

We also estimated a reverse auto-regressive causation path model because it could be argued that individuals who reported more depressive symptoms and/or who were prescribed an antidepressant treatment might be less likely to volunteer (see also the assessment of baseline differences). In this path model, we predicted one's voluntary activity based on one's depressive symptoms and being prescribed an antidepressant treatment, both cross-sectional and longitudinal.

## Results

Prior to presenting the results, we present (1) a comparison of those who volunteered versus those who did not volunteer at the start of the study (thus at T1) in terms of the above outlined control variables, symptoms of stress, and the experience of depressive symptoms at the start of the study, (2) a comparison of those who volunteered less than the mean number of hours (i.e., < 5 h per week), the mean number of hours (i.e., between 5 and 6 h per week), and more than the mean number of hours (i.e., > 6 h per week) in terms of the above outlined control variables, symptoms of stress, and the experience of depressive symptoms at the start of the study, and (3) the correlations between all key variables.

*For the first comparison*, we found statistically significant differences between volunteers and non-volunteers at T1 for (1) age (volunteers were significantly older than non-volunteers; *t* = 8.94, p < .001, mean difference = 3.04), (2) general health (volunteers had a significantly higher general health than non-volunteers; *t* = 9.90, *p* < .001, mean difference = .23), (3) symptoms of stress (volunteers had a significantly lower experience of symptoms of stress than non-volunteers; *t* = 7.73, *p* < .001, mean difference = .10), and (4) the experience of depressive symptoms (volunteers had a significantly lower experience of depressive symptoms than non-volunteers; *t* = 8.67, *p* < .001, mean difference = .20).

*For the second comparison*, we found statistically significant differences between the three voluntary work groups at T1 for (1) age (the group that volunteered less than the mean number of hours was significantly younger compared to the group that volunteered the mean number of hours; *t* = 2.18, *p* = .031, mean difference = 1.63, and the group that volunteered more than the mean number of hours; t =2.13, *p* = .034, mean difference = 1.19), and (2) gender (the group that volunteered more than the mean number of hours was comprised of significantly more males compared to the group that volunteered less than the mean number of hours; *t* = 6.41, *p* < .001, mean difference = .16, and the group that volunteered the mean number of hours; *t* = 3.04, *p* = .003, mean difference = .13)^2^

### Descriptive statistics

Table 1 provides an overview of the means, standard deviations, and correlations of all variables under study.

**Table 1:** Descriptive statistics and correlations matrix.

Variable	Mean	SD	Range	1	2	3	4	5	6	7	8	9	10	11	12	13
1. Age (T1)	60.49	11.42	24-74	-												
2. Gender (T1)	.57	-	-	-.19⁎⁎⁎	-											
3. General health (T1)	2.07	.88	1-5	-.11⁎⁎⁎	.03	-										
4. Stress (T1)	1.76	.46	1-4	-.31⁎⁎⁎	.14⁎⁎⁎	-.02	-									
5. Stress (T2)	1.77	.50	1-4	-.31⁎⁎⁎	.15⁎⁎⁎	-.03	.66⁎⁎⁎	-								
6. Stress (T3)	1.75	.45	1-4	-.30⁎⁎⁎	.15⁎⁎⁎	-.01	.60⁎⁎⁎	.63⁎⁎⁎	-							
7. Depression (T1)	1.69	.80	1-4	-.26⁎⁎⁎	.11⁎⁎⁎	-.01	.77⁎⁎⁎	.58⁎⁎⁎	.54⁎⁎⁎	-						
8. Depression (T2)	1.76	1.06	1-5	-.27⁎⁎⁎	.10⁎⁎⁎	-.03	.58⁎⁎⁎	.58⁎⁎⁎	.47⁎⁎⁎	.63⁎⁎⁎	-					
9. Depression (T3)	1.74	.83	1-5	-.23⁎⁎⁎	.11⁎⁎⁎	-.05*	.54⁎⁎⁎	.54⁎⁎⁎	.77⁎⁎⁎	.61⁎⁎⁎	.50⁎⁎⁎	-				
10. Volunteer (T1)	5.97	7.27	0-99	.11⁎⁎	-.15⁎⁎⁎	-.04	-.06	-.06	-.08*	-.10⁎⁎	-.08*	-.09*	-			
11. Volunteer (T2)	5.77	6.19	0-70	.09⁎⁎⁎	-.12⁎⁎⁎	.01	-.04	-.05⁎⁎	-.07⁎⁎⁎	-.05⁎⁎	-.04*	-.08⁎⁎⁎	.67⁎⁎⁎	-		
12. Volunteer (T3)	5.78	6.14	0-81	.08⁎⁎⁎	-.10⁎⁎⁎	.02	-.04	-.05⁎⁎⁎	-.05⁎⁎⁎	-.04*	-.04*	-.07⁎⁎⁎	.62⁎⁎⁎	.70⁎⁎⁎	-	
13. Anti-depression treatment (end study)	.21	.41	0-1	-.09⁎⁎⁎	.13⁎⁎⁎	-.01	.30⁎⁎⁎	.30⁎⁎⁎	.32⁎⁎⁎	.32⁎⁎⁎	.27⁎⁎⁎	.33⁎⁎⁎	-.01	-.04⁎⁎	-.04*	-

Notes:

⁎ *p* < .05;

⁎⁎ *p* < .01,

⁎⁎⁎ p ≤ .001. *N*_T1_ = 6,542 ; *N*_T2_ = 6,797 ; *N*_T3_ = 3,987 ; The reported means for gender and antidepressant treatment refer to the percentage of respondents who indicated that they were female and were prescribed an antidepressant treatment, respectively. Stress refers to symptoms of stress.

### Inferential statistics

**Fig. 2:**
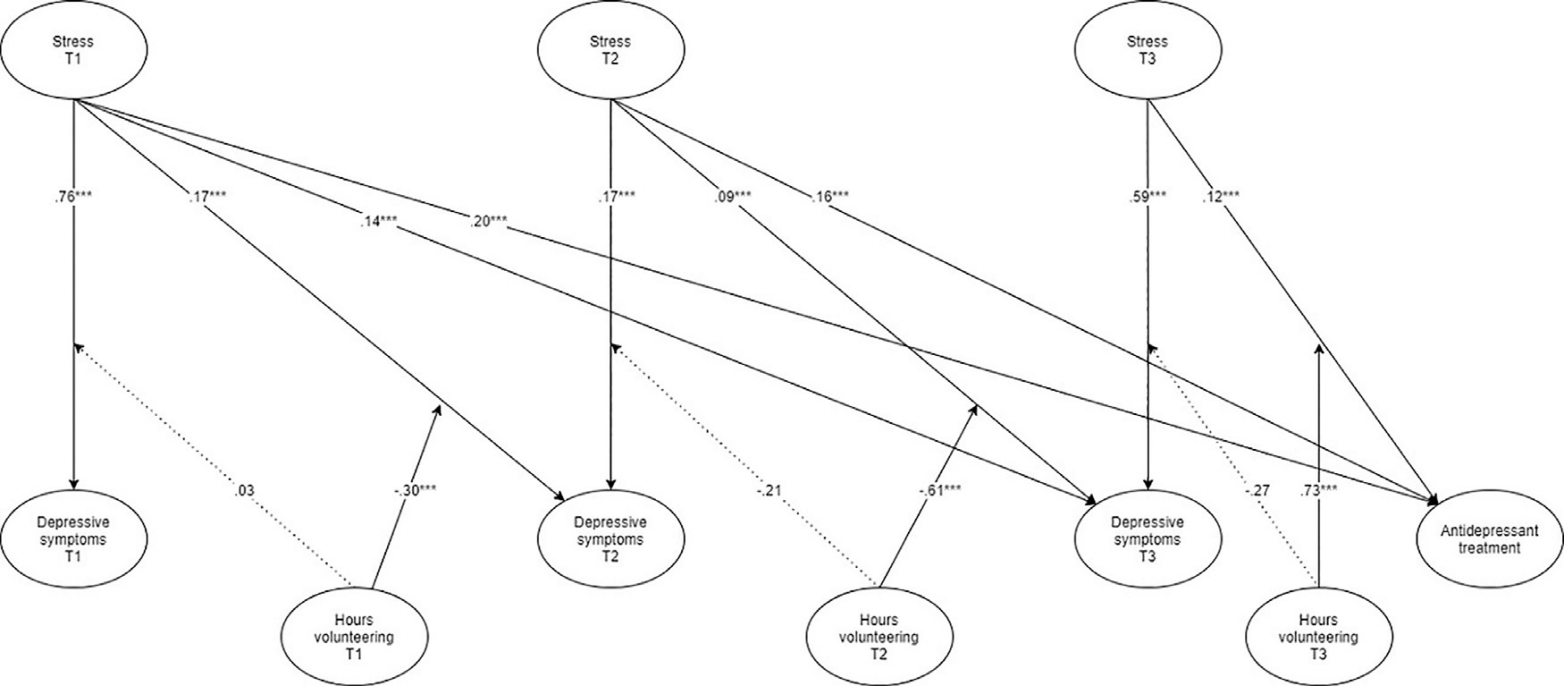
Standardized estimated paths in the longitudinal auto-regressive path analysis.

Notes: * *p* < .05; ^⁎⁎^*p* < .01, ^⁎⁎⁎^ p ≤ .001. Dotted lines indicate non-significant relationships. Although auto-regressive paths were excluded for ease of interpretation, results indicate change in each variable by including the auto-regressive effect between a variable and the same variable at the previous point in time. Stress refers to symptoms of stress.

**Fig. 3:**
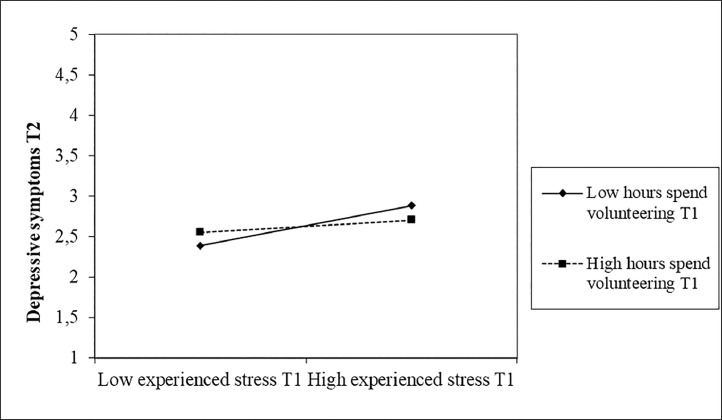
Moderation of hours spent volunteering at T1 on the relationship between symptoms of stress at T1 and depressive symptoms at T2.

*Normal auto-regressive path model*. Our standardized results (see also Fig. 2 for a graphical representation) indicate that (1) symptoms of stress at T1 were positively related to depressive symptoms at T1 (β = .76, SE = .01, *p* < .001), T2 (β = .17, SE = .02, *p* < .001), and T3 (β = .14, SE = .02, *p* < .001), (2) symptoms of stress at T2 were positively related to depressive symptoms at T2 (β = .17, SE = .02, *p* < .001), and T3 (β = .09, SE = .02, *p* < .001), and (3) symptoms of stress at T3 were positively related to depressive symptoms and T3 (β = .59, SE = .02, *p* < .001). These results support the cross-sectional and longitudinal positive associations between symptoms of stress and self-reported depressive symptoms (R^2^_depressive symptoms T1_ = .57; R^2^_depressive symptoms T2_ = .60; R^2^_depressive symptoms T3_ = .61). Moreover, we found that individuals who reported more symptoms of stress at T1, T2, and T3 were 1.64 (95%CI [1.46;1.91]), 1.49 (95%CI [1.24;1.74]), and 1.40 (95%CI [1.21;1.60]) times more likely to be prescribed an antidepressant treatment, respectively (R^2^_antidepressant_ _=_ .21). With respect to the moderating role of voluntary work, we found that the number of hours spent volunteering at T1 did not moderate the relationship between symptoms of stress at T1 and depressive symptoms at T1 (β = .03, SE = .09, *p* = .810), whereas the number of hours spent volunteering at T1 did moderate the relationship between symptoms of stress at T1 and depressive symptoms at T2 (β = -.30, SE = .09, *p* < .001). Examination of the interaction plot (see Fig. 3) by means of a simple slope analysis (thus including -1SD, mean, and +1SD^3^) revealed that under low (-1SD; *t* = 7.74, *p* < .001) and mean (*t* = 2.45, *p* = .015) levels of volunteering the relationship between symptoms of stress at T1 and depressive symptoms at T2 was significantly stronger than under high levels of volunteering (+1SD).

**Fig. 4:**
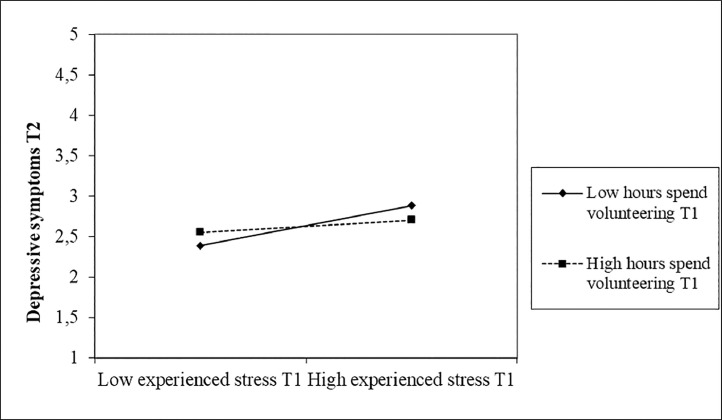
Moderation of hours spent volunteering at T2 on the relationship between symptoms of stress at T2 and depressive symptoms at T3.

Next, we found that the number of hours spent volunteering at T2 did not moderate the relationship between symptoms of stress at T2 and depressive symptoms at T2 (β = -.21, SE = .14, *p* = .120), whereas the number of hours spent volunteering at T2 did moderate the relationship between symptoms of stress at T2 and depressive symptoms at T3 (β = -.61, SE = .15, *p* < .001). Examination of the interaction plot (see Fig. 4) revealed that under low (-1SD; *t* = 3.94, *p* < .001) levels of volunteering the relationship between symptoms of stress at T2 and depressive symptoms at T3 was significantly stronger than under mean or high levels of volunteering (+1SD).

In addition, we found that the number of hours spent volunteering at T3 did not moderate the relationship between symptoms of stress at T2 and depressive symptoms at T3 (β = -.27, SE = .13, *p* = .05). In sum, these findings support the longitudinal—but not the cross-sectional—mitigating effect of voluntary work. Moreover, we also found that the number of hours spent volunteering at T3 moderated the relationship between symptoms of stress at T3 and the likelihood of been prescribed an antidepressant at T3; with respondents with high levels of volunteering being 1.14 (95%CI [1.05;1.26]) times less likely to be prescribed an antidepressant; supporting the cross-sectional—but not the longitudinal—mitigating effect of voluntary work.

*Reversed auto-regressive path model.* Although the reverse auto-regressive path model did not fit our data, as indicated by a 95% confidence interval for the difference between the observed and the replicated chi-square values that included zero (95% CI [-34.56; 30.99], *p* = .55), we nonetheless deem it important to present the significant associations in this model. Our standardized results indicate that depressive symptoms at T1 were negatively related to the number of hours spent volunteering at T1 (β = -.10, SE = .05, *p* = .023). Moreover, we found that depressive symptoms at T2 were negatively related to both the number of hours spent volunteering at T2 (β = -.08, SE = .03, *p* = .015), and the number of hours spent volunteering at T3 (β = -.06, SE = .03, *p* = .015). These findings indicate that, despite the lack of support for this reverse auto-regressive path model, that the experience of depressive symptoms at T1 and T2 may reduce the number of hours spent volunteering at the same point in time (T1 to T1 and T2 to T2) and over time (T2 to T3).

## Discussion

The current study aimed to investigate the role of symptoms of stress as a risk factor in the onset of depressive symptoms and the ultimate likelihood of being prescribed an antidepressant following a medical diagnosis, akin to introducing voluntary work as an intervening mechanism to break down this unfavorable association. In doing so, our work contributes to a very small body of extant literature which has focused on the potential stress-buffering effects of voluntary work by demonstrating that voluntary work (more hours of voluntary work) moderates the relationship between (1) symptoms of stress and self-reported depressive symptoms over time, and (2) symptoms of stress and the likelihood of being prescribed an anti-depression treatment following a medical diagnosis.

### Discussing the results and their implications

Consistent with the Stress Reaction Model and the Accumulation Model (Ford et al., 2014; Pindek et al., 2019; Zapf et al., 1996), we indeed found that symptoms of stress are positively associated with depressive symptoms both at the same point in time (cross-sectional), as well as over time (longitudinal) and ultimately with the development of major depression and thus the likelihood of being prescribed an antidepressant following a medical diagnosis. These results thus reiterate previous findings that the experience of symptoms of stress at a single point in time (evidence for the cross-sectional relationship; e.g., Cheung & Yip 2015; Debowska et al. 2020; Jones-Bitton et al. 2020; Sameer et al. 2020; Shen et al. 2014; Varma et al. 2021) and the accumulation of symptoms of stress over time (evidence for the longitudinal relationship; e.g., Gao et al. 2020; Hosang et al. 2010, 2012a; Huang et al. 2018; Paulson 2022; Planchuelo-Gómez et al. 2020; Probst et al. 2020) may trigger self-reported depressive symptoms using a validated multi-item scale while simultaneously demonstrating the importance of also relying on more objective indicator of major depression such as being prescribed an antidepressant following a medical diagnosis.

Second, and more importantly, in line with the proposed mitigating effect of voluntary work, we found evidence for the modest beneficial effects of voluntary work in breaking down the positive stress-depression relationship. Our findings indicate that voluntary work is a long-term antidote to the experience of depressive symptoms and, ultimately, major depression following a medical diagnosis. That is, it seems that hours spent volunteering indeed alleviates depressive symptoms in the long-term (longitudinal) and the likelihood of being prescribed an antidepressant following a medical diagnosis in the face of symptoms of stress. This means that voluntary work has long-lasting benefits when it comes to reducing self-reported depressive symptoms, as well as reducing the likelihood of being prescribed an antidepressant following a medical diagnosis. Overall, these findings are important for voluntary organizations. More generally, these results contribute to a growing attention for the well-being and health benefits associated with voluntary work (for a review see Anderson et al. 2014; see also Bowe et al. 2022; Burr et al. 2011; Griep et al. 2015, 2017; Kamerāde & Bennett 2018; Li & Ferraro 2005; Mak & Fancourt et al. 2022; O'Brien et al. 2010; Pillemer et al. 2010; Russell et al. 2022). However, we did not find evidence for the cross-sectional mitigating effect of voluntary work in the sense that hours spent volunteering did not moderate the relationship between symptoms of stress and depressive symptoms at the same point in time. The absence of these cross-sectional relationships could be (partially) explained by the argument that the mitigating effect of voluntary work take time to develop and that the beneficial effects of voluntary work as tempering symptoms of stress do not happen instantaneous but rather only when individuals engage in voluntary work over time; a finding that parallels recent research findings in other domains such as the accumulated, but not the immediate, effect of perceptions of job insecurity on stress-related mechanisms when predicting mental health concerns (Griep et al., 2021).

Finally, an important implication to discuss pertains to the fact that mental health benefits from volunteering may vary by age. This is an important note to make given the relatively high average age of our respondents. In line with Activity Theory (Lemon et al., 1972; Longino & Karl, 1982), volunteering should have greater benefits (i.e., larger mitigating effect of voluntary work) for older adults because they are traditionally more engaged in voluntary work and thus may benefit more from the protective effect of voluntary work. In addition to variance in the time spent volunteering, the motives to volunteer may also shift by age. That is, while most young to middle-aged people tend to volunteer as an extension of their work and family roles (Musick & Wilson, 2003), older people often engage in voluntary work as a way to replace the loss of previous work and/or family roles. As a consequence of these different motives, younger volunteers often experience their voluntary activities as less discretionary compared to their older counterparts for whom voluntary work is an ideal way to combine a discretionary activity of leisure and work with social contact. Voluntary work may thus hold a more central place in the lives of older volunteers compared to their younger and middle-aged counterparts. As a corollary of these differences, older volunteers may reap greater benefits of their voluntary work; a finding which seems to align with the beneficial effects of voluntary work for retired individuals (Griep et al., 2017).

### Limitations

Some of the study's limitations need to be taken into consideration. First, although we used well-established scales to measure symptoms of stress and depressive symptoms, the self-rated nature of these variables may pose a number of methodological concerns regarding common method variance and response bias (Podsakoff et al., 2012). Moreover, the use of self-reported measures in longitudinal designs, such as the one used in this study, may also pose problems with regard to (1) ceiling and floor effects (where people reporting the highest or lowest level of experienced stress or depressive symptoms cannot report a subsequent improvement or deterioration), and (2) stability in symptoms of stress or depressive symptoms even when change in underlying stress or health is occurring (Gunasekara et al., 2012). We largely overcame this issue through the inclusion of more objective measures of depression (being prescribed an antidepressant treatment following a medical diagnosis) and by separating our variables over time.

Finally, it is unlikely that those who decided to engage in voluntary work constitute a random sample of all respondents. Those who engaged in voluntary work may have different personalities or motives to engage in voluntary work versus those who choose not to engage in voluntary work; which may have impacted the relationship between symptoms of stress and depression. In line with the work by Mowen & Sujan (2005), it would be advisable for future studies to include measures of altruism, the need for activity and learning, and the motive to help because these authors have shown that these traits were important predictors of one's engagement in voluntary work.

### Future research directions

Our findings open up several new avenues for research. First, although we found evidence for the mitigating role of voluntary work in the relationship between symptoms of stress and depressive symptoms and the likelihood of being prescribed an antidepressant, it is important to note (in line with suggestions made by Griep et al. 2017) that not all voluntary work is created equal. That is, although previous work has demonstrated that the positive well-being and health consequences of volunteering are commonly explained by social and psychological factors, not all types of voluntary work provide the same opportunity to satisfy one's basic psychological needs. Future research could try to further unravel the constellation of voluntary work characteristics that are most likely to be associated with reduced symptoms of stress, and hence with a reduction in depressive symptoms and/or the likelihood of being prescribed an antidepressant following a medical diagnosis.

Second, although this study helps us to understand why formal volunteering may yield well-being and health benefits, it is important to note that formal volunteering is just one of many ways in which individuals can help and support others in their community. Future studies should examine whether similar stress-buffering (and consequently reduced depression) effects present themselves when individuals engage in less formal forms of helping behavior such as informal care for sick or elderly people (Pettigrew et al., 2019; Piatak et al., 2019)

Third, we advise future research to explore potential differences in continuous volunteering (i.e., being a volunteer during all waves of data collection) versus episodic volunteering (i.e., being a volunteer in some but not all waves of data collection) because previous research (e.g., Vallerand, 2000) highlighted that those individuals who are continuously engaged in their voluntary activities might internalize their voluntary activities and hence may experience a stronger stress-buffering effects as the one's found in our study.

### Practical implications

By further unraveling the mitigating role of voluntary work in the relationship between symptoms of stress and the development of depressive symptoms and major depression, the insights from this study support a win-win-win proposition for the individual, the organizations in which these individuals complete their voluntary activities, and the society as a whole. Individuals who spent more hours volunteering when experiencing symptoms of stress, reported fewer depressive symptoms over time and were less likely of being prescribed an antidepressant at the end of the study; both self-reported and objective indicators of depression are associated with considerable psychological suffering, morbidity, and mortality (López et al., 2006; Menken et al., 2000). Moreover, the associated healthcare costs, directly related to depression are estimated to range from anywhere between USD $43,7 billion and USD $83 billion (Patten et al., 2011; Breslau et al., 2008). Our results indicate that it is advisable to promote voluntary work more widely to ensure that people, especially when reporting symptoms of stress, may benefit from the above-described health effects. More specifically, we advise non-profit and non-governmental organization to highlight the well-being and health effects of voluntary work when developing a recruitment and retention policy. Similarly, we advise governmental agencies and policy makers to not only promote the traditional aspects of volunteering (e.g., contributing to society, being altruistic, reducing social isolation), but to also promote the potential individual health benefits (i.e., reducing symptoms of stress and depressive symptoms, reduced likelihood of being prescribed an antidepressant following medical diagnosis). It can be concluded that motiving people, especially when they report symptoms of stress, to volunteer is a promising prescription for one's mental health promotion and maintenance.

## Conclusion

We demonstrated that symptoms of stress are positively associated—both cross-sectional and longitudinal—with the development of depressive symptoms and anti-depression treatment over the course of six years. Moreover, we found support for the protective effect of voluntary work; the number of hours spent volunteering moderated the relationship between symptoms of stress and (1) depressive symptoms over time, and (2) the likelihood of being prescribed an antidepressant at the end of the study. This study underwrites the pivotal role of voluntary work in reducing the impact of symptoms of stress on the development of depressive symptoms and the likelihood of being prescribed an antidepressant.

## Data availability statement

The data used in this manuscript is protected under The Personal Data Act and all data are bound to secrecy, meaning that the data cannot be copied or disclosed on open science platforms such as the Open Science Framework. However, you can request the data used in this manuscript from any of the authors on this manuscript; all authors have access to the final data file as used in this manuscript.

## Declaration of Competing Interest

The authors declare that they have no known competing financial interests or personal relationships that could have appeared to influence the work reported in this paper.

## Footnotes

1 In line with the formal definition of voluntary work and the TUS, VWS, WVS, and the ATUS list of voluntary activities, voluntary work in this manuscript included the following categories: “Computer use (volunteer),” “Organizing and preparing (volunteer),” “Reading (volunteer),” “Telephone calls (volunteer, except hotline counseling),” “Writing (volunteer),” “Fundraising (volunteer),” “Administrative and support activities (volunteer),” “Food preparation, presentation, and clean-up (volunteer),” “Collecting and delivering clothing and other goods (volunteer),” “Providing care (volunteer),” “Teaching, leading, counseling, mentoring (volunteer),” “Social service and care activities (volunteer),” “Building houses, wildlife sites, and other structures (volunteer),” “Indoor and outdoor maintenance, repair, and clean-up (volunteer),” “Performing (volunteer),” “Serving at volunteer events and cultural activities (volunteer),” “Participating in performance and cultural activities (volunteer),” “Attending meetings, conferences, and training (volunteer),” “Public health activities (volunteer),” “Public safety activities (volunteer),” “Public health and safety activities (volunteer),” “Waiting associated with volunteer activities,” “Security procedures related to volunteer activities,” “Civic obligations and participation,” and “Waiting associated with civic obligations and participation.

2 Despite these statistically significant baseline differences and differences between the three groups of volunteers, the associated mean differences are to be considered small. Although it is common to assume that such small mean differences between groups hint toward low practical significance, we want to underline the possibility that these mean differences may grow bigger over time (i.e., further widening the gap between those who volunteer and those who do not volunteer). Notwithstanding the above presented significant differences, we wish to underline (1) that we model *change* in depressive symptoms over time, (2) that we control for the above-mentioned control variables while modelling change, (3) that we also tested a reverse auto-regressive path model in which symptoms of stress, depressive symptoms and being prescribed an antidepressant was used as a predictor of voluntary work, and (4) that a systematic review and meta-analysis of the literature on voluntary work in relation to the health and well-being of volunteers could not find clear evidence of the influence of volunteering type and specific voluntary responsibilities on one's health and well-being (Jenkinson et al., 2013).

3 Note that the mean levels of volunteering is not presented in the figure because it is merely used for the simple slope analysis.
